# The Roles of Genetic and Epigenetic Abnormalities in Essential Thrombocythemia

**DOI:** 10.3390/genes17090989

**Published:** 2026-08-24

**Authors:** Dominika Strzała, Wojciech Homenda, Aleksandra Połom, Aleksandra Kellas, Sylwia Bilska, Sylwia Paszek, Natalia Potocka-Wojtowicz, Izabela Zawlik

**Affiliations:** 1 Doctoral School, Faculty of Health Sciences, Pomeranian University, 76-200 Słupsk, Poland; dominika.strzala2000@gmail.com; 2 Department of Hematology and Bone Marrow Transplantation, Provincial Specialist Hospital, 76-200 Słupsk, Poland; wojhom@sl.home.pl (W.H.); aleksandra.polom12@gmail.com (A.P.); aleksandrakellas@gmail.com (A.K.); 3 Department of Physiotherapy and Emergency Medicine, Pomeranian University, 76-200 Słupsk, Poland; 4 Genetics Laboratory, Provincial Specialist Hospital, 76-200 Słupsk, Poland; s.bilska@szpital.slupsk.pl; 5 Laboratory of Molecular Biology, Department of General Genetics, Faculty of Medicine, Collegium Medicum, University of Rzeszow, 35-959 Rzeszow, Poland; spaszek@ur.edu.pl (S.P.); npotocka@ur.edu.pl (N.P.-W.)

**Keywords:** molecular abnormalities, non-coding RNA, DNA methylation, histone modifications, essential thrombocythemia

## Abstract

Essential thrombocythemia (ET) is a Ph(-) myeloproliferative neoplasm characterized by elevated platelet counts. Patients most often have one of the three following driver mutations: *JAK2*, *MPL*, or *CALR*. However, these mutations are not detected in approximately 10–15% of patients, who are thus referred to as “triple-negative” (TN). **Background/Objectives:** In recent years, there have been breakthroughs in the genetic diagnosis of hematological diseases and progress in understanding their epigenetic basis. **Aim:** This study aimed to review the available literature and systematize the current knowledge on the molecular abnormalities in essential thrombocythemia. Particular attention was paid to the impacts of genetic mutations and epigenetic changes—such as abnormalities in non-coding RNA expression, DNA methylation, and histone modifications—on disease development and their clinical significance in patients with essential thrombocythemia. The most important publications on genetic and epigenetic mechanisms in essential thrombocythemia were reviewed and summarized. **Methods:** A review of the available literature was conducted using selected online databases. Publications from 2003 to 2026 were considered, covering the diagnosis and classification criteria for essential thrombocythemia, its genetic and epigenetic basis, and the associated impacts on the disease course. **Conclusions:** Although understanding the precise roles of genetic and epigenetic abnormalities in essential thrombocythemia remains challenging, they offer many opportunities for use as new diagnostic biomarkers and for the development of novel targeted treatment options. Further research is needed to better understand the genetic and epigenetic changes occurring in ET.

## 1. Introduction

Essential thrombocythemia (ET) is the most common cause of primary platelet hyperplasia. It belongs to the group of Ph- myeloproliferative neoplasms and is most often associated with driver mutations in the *JAK2*, *MPL*, and *CALR* genes. Detection of a driver mutation in one of these genes, along with persistent thrombocythemia (platelet count above 450,000/µL) and a characteristic bone marrow finding, constitute the diagnostic criteria for ET according to the 2022 WHO guidelines.

Since the discovery of the *JAK2* mutation in 2005, significant progress has been made in molecular diagnostics and research into the epigenetic basis of the disease. In recent years, many additional possible mutations and abnormalities have been described that may influence the course and prognosis of the disease. This study aimed to review the available literature and systematize the current knowledge on the molecular diagnosis and course of essential thrombocythemia. Particular attention was paid to the impacts of genetic mutations and epigenetic changes—such as non-coding RNA abnormalities, DNA methylation, and histone modifications—on the development of the disease and their clinical significance, and efforts were made to establish a theoretical framework for further research focusing on epigenetic abnormalities in patients diagnosed with essential thrombocythemia.

ET has not received the greatest attention from researchers, primarily due to its rarity and limited impact on patient survival. However, it is important to remember that ET patients still have a reduced quality of life when compared to healthy individuals [1]. The disease can also affect very young people, which should further encourage researchers to seek new diagnostic and therapeutic methods that could improve the quality of life of ET patients [2].

## 2. Materials and Methods

This narrative review was prepared based on inclusion and exclusion criteria, detailed as follows: Publications from 2003 to 2026 covering the diagnosis and classification criteria for essential thrombocythemia; its genetic and epigenetic basis; and the associated impacts on the disease course which were available from selected online databases, such as PubMed, Scopus, and Google Scholar, were considered. English-language papers were identified using specific keywords, including essential thrombocythemia, miRNA, epigenetic disorders, driver mutation, *JAK2*, *CALR*, and *MPL*. Duplicate papers, publications unrelated to the topic, and articles published in a language other than English were excluded. Based on this research, a review and summary of current knowledge on the diagnosis and prognosis of patients with essential thrombocythemia was created, and directions for further research on this disease were proposed.

## 3. Diagnostic Criteria and Risk Stratification

The diagnosis of essential thrombocythemia (ET), according to the current 2022 World Health Organization criteria, requires meeting all major criteria or the first three major criteria and one minor criterion. Major criteria include persistent thrombocythemia (defined as a platelet count of at least 450,000/µL), characteristic bone marrow histopathology (with proliferation of the megakaryocytic lineage and an increased number of large, mature megakaryocytes with multilobed nuclei in the absence of significant enhancement or left shift in neutrophil granulopoiesis and erythropoiesis), and failure to meet the WHO diagnostic criteria for other myeloproliferative and myelodysplastic neoplasms. A further major criterion is the presence of a driver mutation in one of the *JAK2*, *CALR*, or *MPL* genes. Minor criteria include the presence of a marker of disease clonality and no evidence of reactive thrombocythemia [3,4,5,6].

In clinical practice, precise risk stratification of thromboembolic complications is crucial, as it determines the selection of a treatment. The most commonly used tool is the Revised IPSET-Thrombosis Index, a scoring system which incorporates the following variables: age equal to or greater than 60 years, status of the *JAK2* mutation, history of thrombosis, and coexisting cardiovascular risk factors.

In the updated score (2015), *JAK2* mutation and a history of thrombosis are given the greatest weights, as they significantly increase the risk of both a first and a recurrent thrombotic event. It is worth mentioning that only the *JAK2* mutation is included in the score, despite the other mutations having already been described in the literature at the time of the updated score’s publication. Using this score allows for a precise assessment of prognosis and, consequently, the selection of an appropriate management strategy—from monitoring alone to intensive cytoreductive therapy in high-risk patients.

The following methods are used to assess the prognosis in patients diagnosed with ET: the International Prognostic Score for ET (IPSET), as mentioned above, which includes criteria such as age over 60 years, leukocytosis greater than 11,000/µL, and past thromboembolic events [7]; the Mutation-Enhanced International Prognostic Scoring System for Essential Thrombocythemia (MIPSS-ET), which incorporates age over 60 years, leukocytosis greater than 11,000/mL, male sex, and unfavorable mutations (*SF3B1*, *SRSF2*, *U2AF1*, *TP53*); and the triple A survival model (AAA), which takes into account age (separated into two groups—the first is from 50 to 70 years, which is assigned two points; the second is over 70 years old, which is assigned four points), lymphocytosis less than 1700/mL, and neutrophilia greater than 8000/mL [5].

## 4. Driver Mutations

Mutations occurring in essential thrombocythemia, known as ‘driver’ mutations, are often mutually exclusive and initiate the disease without the need for additional mutations. These include genetic alterations in the following three genes: *JAK2* (Janus kinase 2; located on chromosome 9p24), *CALR* (calreticulin; located on chromosome 19p13.2), and *MPL* (myeloproliferative leukemia virus oncogene; located on chromosome 1p34). Each of these alterations influences the continuous activation of the JAK–STAT pathway, leading to the stimulation of thrombopoiesis. Of these, the *JAK2* V617F mutation in exon 14 is the most common (50–60%), the *CALR* mutation is the second most common (20–25%), and the *MPL* mutation is the least common (3–4%). In 10–15% of patients, none of the abovementioned mutations are detected [5,8,9]. Although important in the context of PV, the mutation in exon 12 of the *JAK2* gene is not involved in the pathogenesis of ET [10].

Recent studies have shown that driver mutations can coexist in various combinations; for example, in the study conducted by He et al., coexistence of mutations was observed in 39 of 2651 cases, representing 1.5% of the study participants. The most common combination was *JAK2* V617F/*MPL* (62%), followed by *JAK2* V617F/*CALR* (23%) and *CALR*/*MPL* (*N*  =  5; 13%) [11]. Double-positive mutations are more common when there are fewer abnormal copies of the *JAK2* V617F allele. However, at present, the available evidence is insufficient to determine the impacts of double-positive mutations on the direct phenotypic consequences of the disease [12].

### 4.1. Structure and Impact of JAK2

JAKs are a group of intramembrane nonreceptor tyrosine kinases that mediate signaling from nearly 60 different cytokines and hormones, including thrombopoietin, via the JAK–STAT pathway. Both JAK2 and the other Janus kinases share a similar structure composed of the following four domains: an *N*-terminal domain, FERM (4.1 protein, ezrin, radixin, moesin); an SH2 domain; a pseudokinase domain (JAK homology-2 [JH2]); and a C-terminal kinase domain (JH1). The FERM and SH2 domains mediate binding to the cytoplasmic portion of cytokine receptors. The JH1 domain is the catalytically active portion of the enzyme, which is responsible for substrate phosphorylation. JH2 plays a regulatory role and, importantly, is the site of clinically significant mutations, including *JAK2* V617F.

JAK–STAT activation, as exemplified by the stimulation of thrombopoiesis, occurs through the binding of a ligand—in this case, thrombopoietin—to the hematopoietic cytokine receptor thrombopoietin receptor (TPOR/MPL), which initiates signal transduction. This leads to receptor rearrangement and/or dimerization, followed by autophosphorylation of the JAK2 kinase domain and increased catalytic activity. Activated JAK2 phosphorylates the cytoplasmic tail, enabling the binding of signaling proteins with the SH2 domain, for example, STAT5 in the case of thrombopoiesis. JAK2 phosphorylates STAT5, then dimerizes and is transported to the cell nucleus, where it promotes cell proliferation, differentiation, and survival. JAK2 also activates other pathways involved in cell proliferation and survival, such as the MAPK and PI3K/AKT/mTOR pathways [13,14,15].

Our understanding of the pathogenesis of essential thrombocythemia (ET) significantly advanced in 2005, when the *JAK2* V617F mutation was discovered. This mutation is located in the pseudokinase domain (JH2), specifically in exon 14 on chromosome 9p24. It is a somatic point mutation consisting of a single nucleotide transversion (G1849T). It is the most common driver mutation in ET, occurring in 50–60% of patients [14].

The presence of this mutation is one of the diagnostic criteria for essential thrombocythemia, and is included as a variable in the IPSET-Thrombosis scale [5].

**Figure 1:**
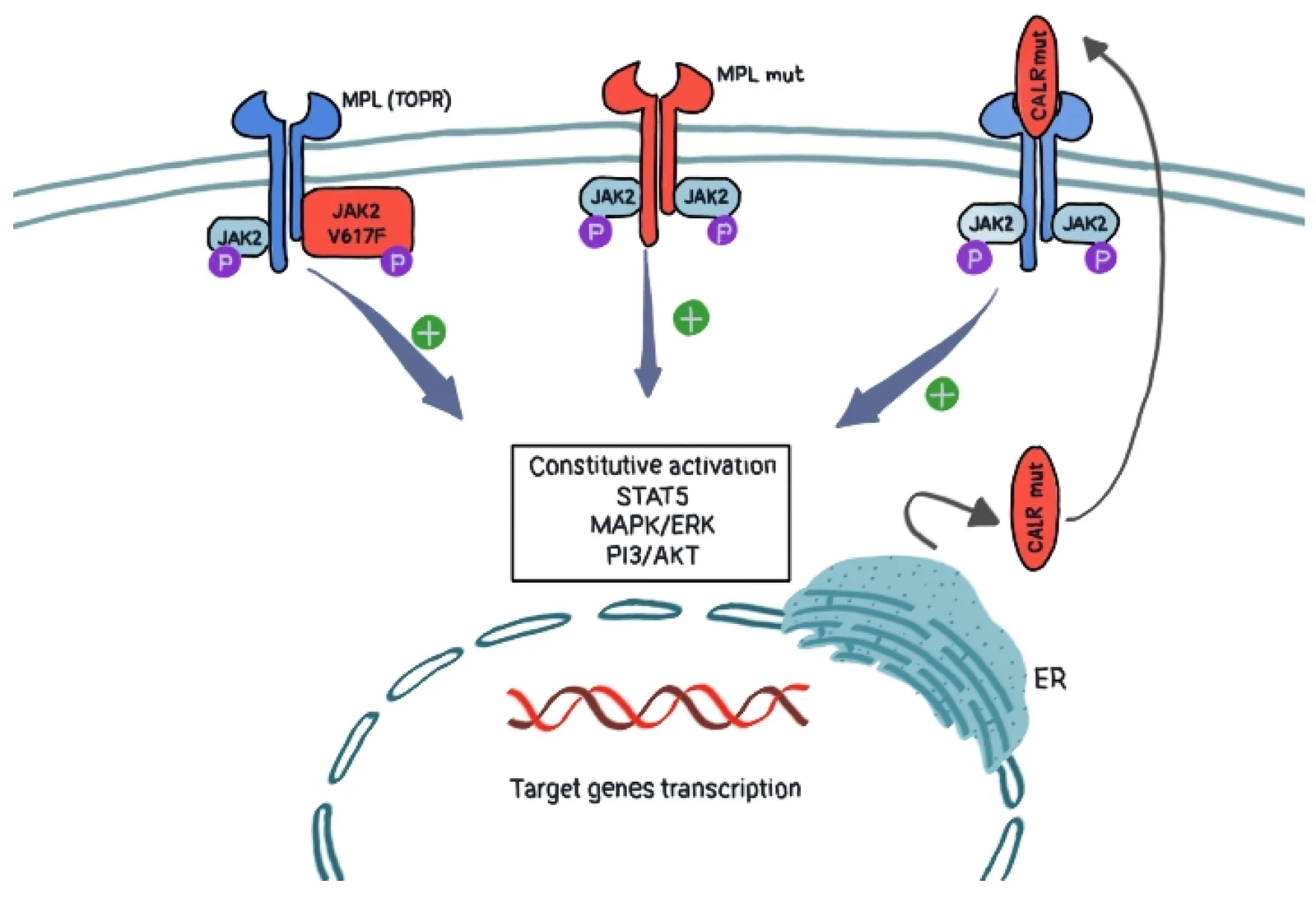
Mutations in the *JAK2*, *CALR*, and *MPL* genes lead to continuous activation of the STAT5, PI3K/AKT, and MAPK/ERK pathways, stimulating the transcription of genes that influence excessive platelet production [14]. Abbreviations: ER—endoplasmic reticulum, mut—mutant.

Analysis of the crystal structure of the JAK2 pseudokinase domain with the *JAK2* V617F mutation revealed spatial changes that inhibit the pseudokinase’s inhibitory function on the kinase domain, leading to continuous JAK2 activation. This results in dysregulation of the JAK/STAT, PI3K/AKT, and MAPK pathways, as well as constant activation of the MPL (or TOPR) receptor, leading to excessive platelet production [14,16,17] (see Figure 1).

Additionally, mutated *JAK2* has been shown to exert epigenetic effects in the cell nucleus. Gene expression changes (among other things) occur through *JAK2* V617F phosphorylation of protein arginine methyltransferase 5 (PRMT5), leading to impaired histone substrate methylation. Recent studies have shown that PRMT5 inhibitors reduce myeloproliferation in cell lines and mouse models, suggesting a potential new therapeutic target [18,19,20].

The *JAK2* mutation increases the expression of proinflammatory cytokines, as evidenced in the literature. For example, Fleischman et al. assessed the correlation between *JAK2* V617F mutation activity and TNFα expression in a 2011 study. They confirmed that an increased number of *JAK2* V617F alleles increases TNFα expression, which in turn promotes clonal cell development [21]. In a 2013 study, Marty et al. demonstrated that mutant JAK2 increases the production of reactive oxygen species (ROS) [22]. According to a recent study conducted in a mouse model, integrin may also play an important role in the regulation of inflammatory cytokines in *JAK2* V617F-positive hematopoietic cells [23]. This might help to confirm the relationship between chronic inflammation and the development of myelofibrosis or the occurrence of thromboembolic complications, demonstrating potential for future prognosis [24,25]. These studies require confirmation in human models and large groups of patients.

A mechanism that may promote thrombosis is the formation of neutrophil extracellular traps (NETs). A recent study has demonstrated that platelets with the *JAK2* V617F mutation can affect neutrophils, inducing NETosis. The same study also suggested that administration of aspirin reduced platelet function and NETosis [20,26].

### 4.2. CALR

The calreticulin (*CALR*) mutation is the second most common driver mutation, occurring in 20–25% of ET patients. It was identified in 2013, through exome analysis of patients without *JAK2* V617F or *MPL* mutations [27].

Calreticulin is an intracellular chaperone protein involved in the control of glycoprotein production and calcium metabolism. There are two main types of *CALR* mutations located in exon 9; in particular, type 1 involves a 52-base pair deletion, while type 2 involves a 5-base pair insertion. These mutations account for over 80% of all *CALR* mutations, with type 1 being more frequently observed in patients with PMF than in those with ET, suggesting a link to the risk of transformation to myelofibrosis. In addition to the mutations mentioned above, over 50 other *CALR* mutations have been described, all involving a single-base-pair frameshift. This results in the formation of an abnormal C-terminal amino acid sequence, KDEL, which usually functions as a signal for protein retention in the endoplasmic reticulum (ER). In the case of mutations, this process is disrupted and the mutated calreticulin is secreted from the ER, thus binding to the thrombopoietin receptor (TOPr/MPL), activating it via dimerization and, as a result, continuously activating JAK2 independent of thrombopoietin [28,29,30,31] (see Figure 1).

Mutated CALR interacts directly with the TOPr receptor and can be secreted from the cell, acting as a rogue cytokine and stimulating cells with TOPr receptors on their surface [32].

Recent studies have indicated that mutant CALR type 1 not only activates TOPr but may also stimulate abnormal megakaryocyte proliferation by activating ERK1/2, thereby activating the MAPK pathway. The Ca^2+^ concentration in the endoplasmic reticulum has also been shown to increase in this context, but the impact of this phenomenon on megakaryopoiesis remains to be studied [33,34].

The peptide resulting from the *CARL* mutation is a neoantigen that, by definition, is not expressed in healthy cells. Therefore, it may be an ideal therapeutic target [35].

### 4.3. MPL

Based on the assumption that the JAK–STAT pathway is crucial to the pathogenesis of myeloproliferative disorders, scientists have sought to discover genetic abnormalities that might influence it. In this regard, an *MPL* (thrombopoietin receptor) mutation was detected in *JAK2* V617F-negative MF patients in 2006, the importance of which in ET patients was subsequently confirmed [36,37]. This is the rarest driver mutation, occurring in 3–4% of patients [9].

*MPL* is encoded on chromosome 1p34 and spans 12 exons. The most common *MPL* W515L receptor abnormality involves a point mutation in exon 10, resulting in a tryptophan-to-leucine change at codon 515. In addition to this mutation, other less common mutations have been described; most are located in exon 10, which encodes the receptor’s transmembrane region. These abnormalities induce constitutive activation of TOPR and, consequently, the JAK–STAT pathway, promoting TPO-independent platelet proliferation [38,39] (see Figure 1).

According to recent reports, cells with mutated MPL exhibit increased cholesterol and lipid metabolism, which may contribute to increased megakaryopoiesis and could become a therapeutic target in the future [40,41].

### 4.4. The Influence of Driver Mutations on the Disease Phenotype

Patients with ET constitute a heterogeneous group, and the course and prognosis of the disease depend primarily on the presence of a specific driver mutation. The most critical phenotypic differences are described below.

A higher median age at diagnosis is characteristic of *JAK2* mutations, whereas a lower median age at diagnosis is characteristic of *CALR* mutations. *JAK2* mutations are more frequently found than *CALR* mutations in women with ET. Peripheral blood counts in patients with *JAK2* mutations show higher hemoglobin and leukocyte levels, while a higher platelet count is characteristic of *CALR* (especially type 2) and *MPL* mutations.

Thromboembolic complications occur more frequently in patients with the *JAK2* mutation. As studies have shown, this mutation increases the risk of both venous thrombosis (especially in younger patients) and arterial thrombosis (in younger and older patients) in an age-dependent manner. In turn, patients with the *CALR* type 2 mutation have a significantly lower risk of thromboembolism when compared to those with the *JAK2* mutation.

The relationships between the presence of specific mutations and MF-free survival have also been analyzed, and it was shown that the *MPL* and *CALR* type 1 mutations—especially the former—increase the risk of progression. It was also demonstrated that these mutations are associated with a higher risk of fibrosis progression. In contrast, the *JAK2* mutation was associated with a lower risk of fibrosis.

A higher rate of serious bleeding events was observed in patients with the *MPL* and *JAK2* mutations, when compared to patients with the *CALR* mutation [5,28,42,43].

Differences in disease phenotype determined by specific driver mutations may allow for more precise patient categorization and more personalized care. Attention to genetic changes occurring in a given patient may facilitate the detection of an increased risk of thromboembolism or progression, thus increasing vigilance in patient follow-up.

A summary of the mechanism and impact on the disease phenotype of driver mutations is provided in Table 1.

### 4.5. Variant Allele Frequency

With the introduction of high-resolution diagnostic techniques, such as NGS, it has become possible to evaluate large genome samples at once, thus quantifying the rate of occurrence of a given mutation, which can be reported as the variant allele frequency (VAF). Abnormalities are then described as their proportion in the tested sample. Research to determine the relationships between VAF values, disease phenotypes, and therapeutic responses is ongoing [44]. Importantly, mutations with a low VAF are most often somatic, while those with a VAF close to 50% may have a germline origin [45].

The potential impact of increased *JAK2* V617F VAF on an increased risk of progression to myelofibrosis in patients with polycythemia vera has been initially demonstrated. In the study by Loscocco G. G. et al., a cutoff point for *JAK2* V617F VAF above 35% was established, above which the risk of myelofibrotic progression increases in patients with essential thrombocythemia [46]. Other authors have also proposed a cutoff point for *CALR* mutations, where the risk of progression from ET to MF is increased when the VAF exceeds 60% [5].

Tashkandi H. et al. presented a cutoff point for *JAK2* V617F VAF of 2%, above which the development of myeloproliferative neoplasm may occur. Furthermore, it was noted that an elevated VAF—particularly in the case of the *JAK2* V617F mutation—characterizes cases of patients with more pronounced abnormalities in blood counts and a higher risk of thromboembolism, whereas an elevated VAF in the *CALR* mutation indicates a more aggressive form of the disease and a poorer prognosis [47].

In the study by Guglielmelli P. et al., *JAK2* VAF was analyzed in 165 patients with ET and it was shown that the mean VAF was 24%, significantly lower than that reported in patients with PV (52%) and MF (61%) [48].

Brown R. assessed the molecular response in the form of VAF reduction in patients with MPN treated with PegIFNα. A response was observed in 91% of patients, with no complete response defined as a reduction in *JAK2* V617F VAF below 2%. Of the subjects who achieved a response, 50% achieved a minor molecular response and 50% achieved a partial molecular response, defined as a reduction in baseline VAF by 50% or more and a reduction of 20–49%, respectively. In this group of patients, the mean VAF reduction following PegIFNα therapy was 42.3% [49].

Ng Z. Y. et al. assessed the responses of patients with *MPN* to treatment with interferon (IFN), anagrelide (ANA), and ruxolitinib (RUX), resulting in a reduction in the VAF of the *CALR* mutation. The study demonstrated that most patients achieved a molecular response, reducing VAF by 20–50% from baseline within 1–2 years of therapy. Although similar results have been previously reported for IFN therapy, the cited publication demonstrated that a molecular response was also achieved in nearly one-third of patients treated with RUX. In contrast, an increase in VAF was observed in patients receiving ANA therapy [50].

It is important to remember that, despite the proven ability to achieve a complete molecular response with IFN therapy, some factors contribute to poorer treatment outcomes; for example, *IFNL4* gene polymorphism has a proven impact on molecular response, patients with the *CALR* mutation respond less well to IFN therapy than those with the *JAK2* V617F mutation, and the presence of additional mutations (e.g., *TET2* or *DNMT2A*) may reduce the response to IFN [45].

VAF assessment is still not widely available in many centers. Future consideration should be given to standardizing this parameter when assessing treatment response in ET patients, which may enable the rapid identification of patients who are losing response and thus accelerate the process of changing treatment in those who require it. This may be particularly useful in patients with mutations that limit treatment response.

## 5. “Triple-Negative” Patients

Among patients with myeloproliferative neoplasms, a significant group of patients presents a considerable diagnostic challenge. These include so-called “triple-negative” (TN) patients, in whom none of the previously described target mutations can be detected. TN patients are most often described in the context of essential thrombocythemia. In a meta-analysis by Reddy et al. including 623 patients with myeloproliferative neoplasms lacking target mutations, 362 were diagnosed with ET. Interestingly, the study group included so-called “truly triple-negative” patients, in whom no other MPN-related mutations were detected on NGS. Most of these patients were women and had essential thrombocythemia. The remainder had various epigenetic abnormalities and splicing mutations [51].

In the study conducted by Cattaneo et al., TN-ET was shown to be a relatively slowly progressive, indolent disease entity. During a 32-year follow-up of a cohort of 40 patients, only two experienced thrombotic episodes and no cases of progression of myelofibrosis or leukemic transformation were detected. One patient experienced disease progression to myelodysplastic syndrome, suggesting the need for extensive diagnostic workup during patient monitoring [52].

Another study characterized platelets in patients with TN-ET compared with those with *JAK2* V617F. Patients without the mutation were younger and experienced fewer thrombotic events. This phenomenon was explained through a comparison of platelets between groups; in particular, platelets from TN patients had—among other things—significantly reduced CD62P expression; slightly reduced calcium levels; increased mitochondrial membrane potential; and, most importantly, reduced ROS levels compared to platelets isolated from patients with the mutation. This last difference may contribute to reduced platelet activation and, consequently, a reduced risk of thrombosis in TN patients [53].

In a large study conducted at the Mayo Clinic involving 1000 patients diagnosed with essential thrombocythemia, 8% of the patients had triple-negative status, with karyotype abnormalities detected in 4% of them. The median age in this group was 50 years, and 73% of the patients were women. The gender distribution of patients was similar to that of the *JAK2* mutation group, but the median age was significantly higher in the *JAK2* mutation group. A difference was also observed in blood count parameters; namely, TN patients had lower hemoglobin and leukocyte counts, but higher platelet counts. The comparison between TN patients and those with the mutated *CALR* gene is somewhat different, in that the latter group consisted mostly of men with higher hemoglobin concentrations, but with a lower history of arterial and venous thrombosis and fewer serious bleeding events. Among all patients studied, 8% experienced a major bleeding event before diagnosis, with the highest incidence observed in TN patients, at 13%. Post-diagnosis bleeding events occurred in TN patients at 9%—similar to those in patients with *CALR* mutations, but lower than those associated with *JAK2* or *MPL* mutations. Triple-negative patients had longer OS than patients with *MPL* or *JAK2* mutations. The risk of myelofibrotic transformation in TN patients was 10% overall, 5% within 10 years, and 19% within 20 years, respectively. Patients with *JAK2* mutations had a similar overall risk, while patients with *CALR* and *MPL* mutations had a higher probability of transformation [51].

Interestingly, the gene expression and DNA methylation profiles determined in TN patients reveal greater similarity to those of patients with driver mutations than to healthy individuals. Increased expression of genes crucial for regulating platelet function, such as *GP6*, *GP1BB*, *ACTN1*, and *ITGA2B*, has also been observed in TN patients. It has been suggested that, in all patients, in addition to activation of the JAK2/STAT pathway, inflammatory signaling pathways such as MAPK, TNF, and NFKappa are also crucial in the pathogenesis of the disease [54]. However, these reports require confirmation and validation in order to better understand the pathogenesis of the disease, particularly in TN patients.

Although the indolent form of the disease is indicated in patients with TN-ET, it is important to remember that diagnostic difficulties in these patients can delay diagnosis and, thus, worsen the prognosis. Diagnostic standards for these patients should be developed based on accurate genetic testing approaches, such as NGS. It is possible that, in the future, the diagnosis of TN patients will be made easier through the development of epigenetic biomarkers.

## 6. Other Mutations

The absence of one of the mutations described above in a given patient does not exclude the presence of other genomic abnormalities. Nangalia et al. demonstrated that the median number of somatic mutations in patients with *BCR-ABL*(-) myeloproliferative neoplasms ranges from 6.5 to 13, comparable to the median number of such mutations in patients with other hematological malignancies but lower than that in patients with epithelial malignancies [55].

Subclonal mutations may be detected in patients with essential thrombocythemia, which most often occur together with driver mutations but may also appear at a different stage of the disease, preceding or developing later than them. In their 2020 article, Accurso et al. noted that there are no established standards for detecting and diagnosing ET based on mutations other than *JAK2*, *CALR*, and *MPL* at present. The presence of non-driver mutations is only one of the minor criteria for the diagnosis of essential thrombocythemia, according to the 2016 WHO recommendation amendment, but performing a next-generation sequencing panel has not yet become a diagnostic standard. The most common subclonal mutations in patients with ET include *DNMT3A*, *TET2*, *ASXL1*, *CBL*, *IDH*, and *IKZF1*, and their precise impacts on the disease course are not yet understood [56].

They likely influence the course of the disease, progression, and leukemic transformation in conjunction with driver mutations. They can be divided, based on their impact on the genome, into those regulating epigenome changes (e.g., *ASXL1*, *TET2*, *EZH2*, *IDH1*, *IDH2*, and *DNMT3A*), those involved in RNA splicing (e.g., *SRSF2*, *U2AF1*, *ZRSF2*, and *SF3B1*), and those affecting transcription (e.g., *TP53*, *IKZF1*, *NF-E2*, and *CUX1*) [5].

Selected mutations are considered unfavorable, as some have been shown to negatively impact the course of the disease and increase the risk of progression. As mentioned earlier, subclonal mutations may precede the emergence of driver mutations in essential thrombocythemia; this includes subclonal mutations in *DNMT3A* or *TET2*, which can shape the disease phenotype. Other mutations, such as *TP53*, *SF3B1*, *U2AF1*, or *EZH2*, have been proven to adversely affect the disease course and shorten the time to leukemic or myelofibrotic progression. Another known mutation that can accelerate myelofibrosis is *ASXL1* [57,58,59].

Interestingly, Tefferi et al. have shown that there are likely no significant differences in the number of associated mutations between patients with and without a specific driver mutation. In the same study, the following associations were noted: the presence of a *TET2* mutation increased the risk of thrombosis and, along with the *SF3B1* mutation, was associated with older age of patients; the presence of an *SF3B1* mutation also resulted in an increased platelet count; and the detection of an *ASXL1* mutation was associated with an enlarged spleen in a given patient [60].

Segura-Diaz et al. have suggested that some mutations—specifically, *DNMT3A*, *TET2*, and *ASXL1*—increase cardiovascular risk through epigenetic regulation, understood in this case as methylation disorders, which lead to increased transcription of proinflammatory genes and, consequently, to the development of atherosclerosis [61]. It seems that providing thromboembolic prophylaxis in TN-ET patients with additional prothrombotic mutations could be a good strategy [62].

In a study involving 183 Mayo Clinic patients diagnosed with essential thrombocythemia, the median survival was 19.9 years. Mutations in *IDH2*, *EZH2*, and *SH2B3* were found to have negative impacts on survival, with the associations with *SH2B3* and *IDH2* remaining significant in multivariate analysis. Mutations in *SH2B3*, *IDH2*, *U2AF1*, *SF3B1*, *EZH2*, and TP53 were found to negatively impact progression-free survival in patients with leukemia or myelofibrosis, with 27 patients harboring one of these mutations. It is important to note that patients with additional mutations were older and more likely to have unfavorable genetic changes. However, after multivariate analysis and the development of a prognostic model, the adverse impacts of these mutations on the disease course remained evident regardless of patient age [63].

It is important to note that the number of mutations accumulating in hematopoietic cells increases with age. It has been estimated that one new mutation arises every 7–8 years, which most often does not lead to disease development. However, some genetic disorders can predispose individuals to cancer, the detection of which is now easier than before thanks to NGS technology. In recent years, two new terms have been coined: CHIP (Clonal Hematopoiesis of Indeterminate Potential), which describes individuals with mutations associated with a predisposition to hematological cancers who do not meet other diagnostic criteria for the disease, and ARCH (Age-Related Clonal Hematopoiesis), which refers to the occurrence of this phenomenon in older adults. CHIP is associated with an increased risk of developing hematological cancers, ranging from approximately 0.5 to 1% per year [5,64].

Luque Paz et al. examined a group of patients with polycythemia vera or essential thrombocythemia to assess the impacts of various mutations on leukemic transformation, and identified which mutations were more likely to lead to disease progression sooner or later after diagnosis. The first group included *IDH1/2*, *RUNX1*, and *U2AF1*, and patients harboring these mutations experienced transformation after an average of three years. Mutations in the *TP53*, *NRAS*, and *BCORL1* genes likely have different impacts, and were associated with leukemia diagnosis at 21 years after the ET or PV diagnosis on average. Interestingly, some mutations were detected in patients already in the chronic phase of the disease, and the number of abnormal allele copies was associated with the time remaining to transformation; namely, the number was lower in patients with late transformation and higher in those with more rapid disease progression [65]. Another study confirmed the theoretical poor prognostic significance of the TP 53 mutation, the presence of which may be associated with a worse response to treatment [66].

In the Mayo Clinic cohort, *SF3B1*, *SRSF2*, and *EZH1* mutations influenced overall survival (OS); *SRSF2*, *EZH2*, and *TP53* mutations influenced leukemia-free survival (LFS); and *SF3B1* and *U2AF1* mutations influenced myelofibrosis-free survival (MFFS). In the Florence cohort, the distribution was slightly different: *SF3B1*, *SRSF2*, and *U2AF1* influenced OS; *TP53*, *U2AF1*, and *RUNX1* influenced LFS; and *SF3B1*, *SRSF2*, and *U2AF1* influenced MFFS [67].

There are also reports suggesting a poorer response to treatment among ET patients harboring additional mutations. This should be taken into account when planning the therapeutic strategy for and monitoring these patients [68]. In the future, it will be necessary to develop new therapeutic methods that provide options for patients with additional mutations that worsen their prognosis [62].

The number of studies confirming the impacts of mutations other than driver mutations on the prognosis and course of ET remains limited. Future research should focus on identifying mutations that, when coexisting with driver mutations, increase the risk of thromboembolism and disease progression. Mutations such as *SF3B1*, *SRSF2*, *U2AF1*, and *TP53* are included in prognostic scales such as MIPSS-ET [69]. It is possible that, in the future, more mutations will be included in prognostic assessments, which can be expected to allow for the recruitment of patients who require increased surveillance. Furthermore, it is important to identify mutations that allow for diagnosis of the disease in TN patients.

Some of the additional mutations mentioned earlier are described in more detail below.

### 6.1. ASXL1

The *ASXL1* gene (additional sex comb-like 1) consists of 13 exons and is located on chromosome 20q11.1 [38]. The protein it encodes plays a role in epigenetic processes and transcriptional regulation by interacting with the polycomb repressive complex (PRC) and transcriptional activators. This gene is present in most hematopoietic cells, and a somatic mutation in its exon 13 leads to a frameshift or nonsense mutation [70].

It has been shown that *ASXL1* mutations can coexist with other mutations in ET patients, including *JAK2* and *TET2*, and sometimes precede their occurrence. Recent studies have reported an association between *ASXL1* mutations and an increased risk of bleeding in patients with MPN [71,72].

As mentioned earlier, the described mutation may accelerate myelofibrosis and worsen the prognosis of patients with ET [73]. More research is needed to determine its possible effects on myelofibrotic or leukemic transformation and explain its exact mechanisms of action.

### 6.2. TET2

*TET2* (Tet methylcytosine dioxygenase 2) belongs to a group of three homologous human proteins, the other two being *TET1* and *TET3*. The *TET2* gene is located on chromosome 4q24 and mutations most often affect its A isoform, which consists of 12 exons. The function of *TET2* is to convert 5-methylcytosine to 5-hydroxymethylcytosine, thereby exerting an epigenetic influence on transcriptional regulation and normal hematopoiesis. *TET2* mutations include frameshift, nonsense, and missense mutations, which occur within several of the 12 exons [38,74,75].

Interestingly, in patients with both *TET2* and *JAK2* mutations, the former was observed earlier in the course of the disease. It has been observed in CD34+CD38- stem cells, demonstrating the presence and impact of this mutation on abnormal hematopoiesis in patients with myeloproliferative neoplasms [76].

### 6.3. CBL

The *CBL* (Casitas B-cell lymphoma) gene consists of 16 exons on chromosome 11q23.3 and encodes a cytosolic protein that inhibits E3 ubiquitin ligase-dependent signaling pathways, including RAS–MAPK and PI3K/AKT. Mutations have been shown to disrupt KIT and FLT3 ubiquitination, leading to oncogenic effects and promoting uncontrolled cell growth. Research in mice has shown that individuals with a defective *CBL* gene have an increased number of hematopoietic stem cells and increased sensitivity to growth factors, as well as splenomegaly. More research is needed to characterize the impacts of *CBL* mutations on the development of myeloproliferative diseases and the potential progression of fibrosis or leukemic transformation [38].

### 6.4. IDH1/2

The *IDH1* gene is located on chromosome 2q33.3 and comprises 10 exons, while *IDH2* is located on chromosome 15q26.1 and comprises 11 exons. They encode isocitrate dehydrogenases 1 and 2, which are NADP+-dependent enzymes that catalyze the oxidative decarboxylation of isocitrate to α-ketoglutarate and convert NADP+ to NADPH. Yonal-Hindilerden et al. have demonstrated that a mutated variant of this genes increases the risk of leukemic transformation in patients with myeloproliferative neoplasms. In another study, Tefferi et al. suggested that *IDH* mutations are common in patients with blast-phase MPN but rare in those with chronic-phase MPN [38,77].

### 6.5. SRSF2

*SRSF2* belongs to the serine/arginine-rich protein family, which is involved in RNA splicing. *SRSF2* mutations involving proline residue 95 (*SRSF2* P95) have been detected in patients with various myeloid neoplasms, including myelodysplastic syndromes, chronic myelomonocytic leukemia, polycythemia vera, essential thrombocythemia, and acute myeloid leukemia. In MPN, *SRSF2* mutations are rarely observed and occur most frequently in patients with MF [78].

At present, the exact role of the *SRSF2* mutation remains unclear. Studies have been conducted in *SRSF2* knock-in mice, concluding that coexpression of the *SRSF2* P95H mutant reduced the number of erythrocytes, neutrophils, platelets, and splenomegaly, but did not induce bone marrow fibrosis in *JAK2* V617F(+) mice [79].

In the few studies published to date in patients with essential thrombocythemia, the *SRSF2* mutation has been associated with a worse prognosis [80,81,82].

### 6.6. SF3B1

Mutations in the *SF3B1* gene are among the most common and, according to previous studies, the most significant spliceosome abnormalities in myeloproliferative diseases. This gene is located on chromosome 2q33.1 and encodes a subunit of the *SF3B1* complex, which is crucial for proper spliceosome function and intron branch site recognition during RNA splicing. Mutations in this gene lead to altered *SF3B1* protein activity, resulting in splicing aberrations and, ultimately, loss of functional protein expression. The impacts of *SF3B1* gene mutations on the development and course of hematopoietic disorders is a subject of ongoing research. As the significance of these mutations is increasingly being uncovered, *SF3B1* analysis is slowly becoming an integral part of hematological diagnostics [63,83,84].

The most important additional mutations occurring in ET patients and their impact on the pathogenesis, phenotype or prognosis of the disease are listed in Table 2.

### 6.7. Future Directions

The roles of many genes in the development and course of ET remain unknown. It is likely that some of them may increase the risk of thromboembolism; for example, higher expression of *F3*, *SELP*, *VEGFA*, and *SLC2A1* in granulocytes and *IL1RAP* in platelets has been observed in ET and PV patients with a history of thromboembolism, when compared to individuals without such a history. The influence of these genes on thromboembolic risk in ET requires further investigation, and may be a fruitful area in the search for targeted therapies [85].

Other genes whose roles in the pathogenesis of ET require further investigation are *LCN2* and *MMP8*, which may influence the survival of megakaryocytes and participate in activation of the inflammatory response [86].

Exploring and discovering the roles of new genes in the pathogenesis and progression of ET may pose challenges and inform future goals for researchers. Any new information about additional mutations occurring in patients could potentially lead to the development of novel biomarkers and targeted therapies.

## 7. Non-Coding RNA

Although just over 60% of the human genome is transcribed, only 2% encodes proteins, with the remainder being converted into microRNAs, long non-coding RNAs (lncRNAs), circular RNAs (circRNAs), and other non-coding RNAs (ncRNAs). It has been shown that ncRNAs can act as regulators of gene expression by influencing chromatin organization, transcriptional regulation, and post-transcriptional processes. This means that ncRNAs are essential for many biological processes; however, they may also contribute to pathophysiological processes, including the occurrence of proliferative diseases [87].

Long non-coding RNA (lncRNAs) are RNA molecules which are longer than 200 nucleotides and do not encode any proteins. These molecules are indispensable in various regulatory processes, such as genomic imprinting, chromatin modification, transcriptional interference, transcriptional activation, X-chromosome silencing, and intranuclear transport [88]. lncRNAs can act as oncogenes in neoplasm development via induction of cell cycle progression, invasion and cell proliferation, anti-apoptosis, and metastasis mechanisms [89].

Bock O. et al. have reported a difference in the expression of IncRNA—specifically, H19—in patients with myeloproliferative neoplasms, who had significantly lower levels of this RNA compared to healthy controls. In patients with ET, an up to six-fold reduction in the expression of this IncRNA was observed [90]. The study described here was published in the early 20th century, and these data have not been thoroughly explained or confirmed in subsequent publications. Therefore, changes in lncRNA expression in ET patients require much more detailed investigations using modern laboratory methods.

circRNAs have a covalently closed loop structure in which the 3′ and 5′ ends are joined together, leaving no free ends and, thus, ensuring greater molecular stability. They are produced by alternative splicing, specifically reverse splicing. This heterogeneous group of molecules participates in the modulation of gene expression by regulating transcription, protein function, and miRNAs [91].

Yu G. et al. showed that, in the bone marrow cells of patients with ET, the expression of one of the circRNAs (i.e., circ_0014614) and caspase3 were reduced when compared to the healthy control group, while the expression of miR-138-5p was increased. In vitro research demonstrated that circ_0014614 influences myeloid cell proliferation and differentiation by influencing the regulation of caspase 3 by miR-138-5p. These changes may promote megakaryocyte proliferation and differentiation via the circ_0014614–microRNA–mRNA axis in ET patients [92].

Wang Q. et al. focused on examining differences in circRNA expression in the bone marrow of individuals with ET compared to a healthy control group. Similarly to the study described above, circ_0014614 was shown to inhibit megakaryocyte differentiation. Furthermore, 1489 circRNAs with increased expression and 1368 with decreased expression were identified in the study group with respect to the control group. The importance of intronic regions—which may be one of the main sources of circRNA in human bone marrow cells—was also described. Further study steps focused primarily on determining the possible impacts of the significantly reduced expression of circDAP3, circASXL1, and circRUNX1, as detected in the studied samples, on the development of ET. It has been shown that miR-29b-1-5p may be a target for circDAP3 and circRUNX1, while miR-34b-5p may be a target for circASXL1 and circRUNX1. These miRNAs may regulate the expression of genes involved in various signaling pathways, such as PI3K and TGF-β, which in turn have been shown to be involved in the pathogenesis of ET [93].

Data characterizing circRNAs in ET are significantly limited. This creates room for further research focusing on understanding the connections between individual circRNAs and other non-coding RNAs. In the future, a detailed understanding of the mechanisms by which individual circRNA–miRNA–mRNA axes influence the pathogenesis of ET may enable the development of highly targeted therapies or specific diagnostic methods.

### miRNA

MicroRNAs (miRNAs) are short, non-coding, single-stranded RNA molecules composed of 18–24 nucleotides that have been shown to regulate biological processes such as cell differentiation, proliferation, and apoptosis. Research has recently been conducted on the roles of miRNAs in many cancers, for example, in gliomas [94].

mRNA is transcribed by polymerase II. Initially, pri-miRNAs are formed, which, through processing by the Drosha complex, become 70–120 nucleotide hairpin structures called pre-miRNAs. In this form, they are transported into the cytoplasm by exportin 5 for processing by the Dicer complex. They become 18–24 nucleotide duplexes, from which—after losing their secondary strand—mature miRNAs are formed. They then enter the RNA-interfering silencing complex (RISC). In this form, miRNAs bind to the 3′UTR of the target mRNA, leading to its degradation or inhibiting protein translation. It has been demonstrated that they can regulate hematopoiesis in both hematopoietic stem cells and progenitor cells. Abnormal miRNA expression has been observed in patients with myeloproliferative neoplasms, in both erythroid cells and circulating granulocytes, platelets, and reticulocytes [95,96,97].

The diverse phenotypes of diseases associated with the *JAK2* V617F mutation may result from distinct miRNA abnormalities. It has been suggested that deregulation of some of these miRNAs may affect the course and prognosis of the disease, regardless of the presence of a specific driver mutation. It has been proven that miRNAs play an important role in hematopoietic cell homeostasis and, therefore, may represent a new therapeutic target for hematological diseases. Creating therapies that target individual miRNAs would be beneficial, given their specificity in interacting with mRNAs responsible for specific stages of cell function. This creates ideal conditions for the development of targeted therapies [98].

In recent years, studies have begun examining abnormalities in specific miRNAs in patients with essential thrombocythemia in comparison to healthy controls. The most important findings are described below.

In the study conducted by Tran et al., the expression levels of several miRNAs in platelets isolated from patients with essential thrombocythemia were compared with those from healthy controls. They found significantly elevated levels of miR-9 and miR-490 in platelets in the study group, while decreased levels of miR-10a, miR-28, miR-126, miR-155, miR-221, miR-222, miR-223, and miR-431 were observed compared to the control group. Furthermore, a positive correlation was observed between miR-431 expression and the platelet parameters MPV (Mean Platelet Volume), IPC (Immature Platelet Count), and IPF (Immature Platelet Fraction). Expression of miR-126 negatively affected platelet aggregation induced by AA (arachidonic acid) or TRAP (thrombin receptor-activating peptide). Furthermore, miR-9 and miR-490 were observed to be negatively correlated with platelet activation and, with respect to driver mutation, their expression was lowest in patients with *JAK2* mutations, which may explain why these patients are more susceptible to thrombotic events than those with *CALR* or *MPL* mutations [99].

In a study by Girardot et al., miR-28 and the related miR-708 and miR-151 were shown to inhibit translation of the MPL receptor and other proteins that are potentially required for megakaryocyte differentiation, such as E2F6 and ERK2. Levels of these microRNAs were also shown to be significantly higher in platelets from patients with myeloproliferative neoplasms—particularly PV and ET JAK2 WT (Wild Type)—compared to healthy individuals. It is known that patients with essential thrombocythemia without *JAK2* mutations have the highest platelet levels, which may suggest a correlation between their growth and elevated miR-28 levels. Increased miR-28 levels are also likely associated with sustained STAT5 activation, leading to the autonomous development of hematopoietic cell lines [100,101].

In the study conducted by Norfo R., the microRNA expression in CD34+ cells collected from the peripheral blood of patients with PMF was compared with that in CD34+ cells from the peripheral blood and bone marrow from healthy controls. The study group demonstrated increased expression of miR-19a-3p, miR-335-5p, miR-379-5p, miR-376c-3p, miR-487b-3p, and miR-494-3p, as well as decreased expression of miR-486-3p, when compared with the control group. The same study demonstrated that overexpression of miR-155-5p—which inhibits the target JARID2—contributes to expansion of the megakaryopoietic lineage, which may influence the PMF phenotype (including hyperplastic megakaryopoiesis) [96].

Tombak A. compared miRNA levels in the peripheral blood of patients with ET, PV, and PMF with those in healthy individuals, and found higher levels of miR-155 and miR-181a in the study group. While differences in miRNA concentrations were not detected based on the *JAK2* V617F mutation status, it is suspected that the different phenotypes of all diseases occurring with this mutation (i.e., ET, PV, and PMF) may depend on epigenetic changes, including the influence of individual miRNAs [102].

In 2014, Benati M. et al. reported an increased level of miR-143 in granulocytes of patients with myeloproliferative neoplasms compared with a healthy control group, where this increase was positively correlated with a higher number of mutant *JAK2* V617F gene copies [103].

In a study by Ferrer-Marin et al., miR-146a—which is involved in inflammatory processes—was shown to be associated with the progression of fibrosis in patients with myeloproliferative neoplasms by modulating NF-κB signaling through its effects on TRAF6 and IRAK1. Furthermore, lower levels of miR-146a were shown to positively correlate with increased STAT3 activity in the myeloid cell population. miR-146a, as an independent risk factor for faster transformation to MF, may thus serve as a specific biomarker for monitoring progression [104].

Other miRNAs found to be dysregulated in ET patients include miR-34a, miR-342, miR-326, miR-105, miR-149, and miR-14. Atypical, ectopic expression has also been observed in miR-10a and miR-150. All of these contribute to the hematopoietic proliferation and differentiation characteristic of ET, particularly in the megakaryocytic lineage [105].

In 2016, Navarro et al. compared the microRNA profile in platelets from individuals with ET to that of a healthy control group. A link was demonstrated between individual microRNAs and the regulation of key ET genes—specifically, the influence of miR-221 on *SOCS1*, miR-221 on *SOCS3*, miR-203 on *SOCS3*, and miR-23a on *PTPN11*. The study identified 70 different microRNAs, the expression of which differed between platelets from healthy and affected individuals. Differences in microRNA expression were also described between patients, depending on the driver mutation they possessed; notably, the levels of miR-15a, miR-150, and miR-519a differed. In patients without the *JAK2* V617F mutation, negative correlations were found between miR-203 and miR-221 and between *SOCS1* and *SOCS3*, both of which inhibit the JAK/STAT pathway, which is crucial in the pathogenesis of ET. Therefore, increased levels of these microRNAs in patients without the *JAK2* mutation may be essential in activating signaling pathways that are critical in the pathogenesis of the disease [106].

Yin L. et al. described a relationship between the decreased levels of miR-375 observed in patients with MPN and the abolition of JAK2/STAT pathway inhibition, indicating that reduced expression of this microRNA in patients with essential thrombocythemia contributes to constitutive JAK2/STAT activation. Elevating miR-375 levels in patients with MPN to those observed in healthy individuals may thus represent a new therapeutic target, for example, through the use of histone deacetylase (HDAC) inhibitors, which restore miR-375 expression [107].

Conclusive conclusions regarding the associations between individual miRNAs and driver mutations are often lacking. miR-125 potentially plays a role in the pathogenesis of ET, but data regarding its relationship with the presence of *JAK2* mutations remain unclear and require confirmation in further studies [108,109].

A good example of the interplay between epigenetic factors and mutations in ET is the association of miR-101 with SOCS2 inhibition and constitutive activation of the JAK2 signaling pathway—which, in a positive feedback loop, drives miR-101 synthesis. More studies demonstrating similar relationships and their effects on the course of ET are needed, which may help us to better understand the pathomechanisms underlying the disease and allow for the identification of new drug targets [110].

Regarding the impacts of miRNAs on the pathogenesis and course of ET, many molecules require further investigation. These include miR-1268a, which has been reported to interact with eight genes involved in platelet formation and activation, namely, *CDH6*, *EHD2*, *FUT1*, *KIF26A*, *LINC00346*, *PTPRN*, *SERF1A*, and *SLC6A9* [111]. However, more studies in larger patient groups are needed to confirm these reports.

Another interesting process relates to the changes in miRNA expression observed during therapy in patients with ET. The levels of miR-32, miR-31, miR-101, and miR-1275 detected during ruxolitinib therapy may indicate the roles of these miRNAs as therapeutic targets. However, the precise mechanisms by which these changes occur remain unknown and require further study [54].

Epigenetic abnormalities, including changes in the expression of individual miRNAs, may interact with driver and associated mutations, influencing the pathogenesis and progression of ET. The most important associations and their impacts on the disease course are summarized in Table 3.

## 8. DNA Methylation

DNA methylation is an epigenetic process that occurs in mammals through the transfer of a methyl group to the C5 position of cytosine, creating 5-methylcytosine. This process regulates gene expression, specifically inducing the action of repressive proteins affecting individual genes or inhibiting the binding of transcription factors to DNA. The methylation profile of individuals changes during development through de novo demethylation and methylation processes. Ultimately, a stable, unique methylation pattern is established, which regulates transcriptional processes in cells of specific tissues [112]. Methylation—leading to so-called gene silencing—may also affect tumor suppressor genes, the inhibition of transcription of which may lead to carcinogenesis [113].

In recent years, efforts have been made to examine the DNA methylation profiles of patients with MPN. It has been demonstrated that patients with PV, ET, and MF exhibit altered methylation profiles compared with healthy controls. Although individual types of Ph(-) myeloproliferative neoplasms did not differ significantly from each other, an increase in differentially methylated regions was observed in patients who transformed to AML compared to those in the chronic phase. This may suggest that altered DNA methylation is involved in disease progression [114].

In their study, Zhou J. et al. focused on examining differences in the activity of a negative regulator of the JAK/STAT pathway—namely, the *PTP1B* gene—in patients diagnosed with MPN compared to healthy controls. They identified differences in the frequencies of seven single nucleotide polymorphisms (SNPs) in the coding region of this gene and aberrant hypermethylation of its promoters in patients with MPN, compared to healthy controls. These results suggest that patients with myeloproliferative neoplasms—including essential thrombocythemia—may have abnormalities in the *PTP1B* gene, which could influence the pathogenesis of these diseases [115].

Fourouclas N. et al. examined the prevalence of hypermethylation of the *SOCS1* and *SOCS3* genes in patients with MPN compared to controls. Hypermethylation of the *SOCS3* promoter was detected in 32% of the patients with primary myelofibrosis, suggesting a potential role of *SOCS3* methylation in disease progression. Importantly, similar abnormalities were not confirmed in patients with other conditions, including essential thrombocythemia [116].

A similar comparison of ET patients to a control group has been performed by Födermayr M. et al., examining the methylation of the *SOCS1*, *SOCS3*, and *PTPN6* genes. They demonstrated that patients diagnosed with thrombocythemia had statistically significantly lower *SOCS1* methylation and a tendency toward reduced *SOCS3* and *PTPN6* methylation when compared to healthy individuals. However, no correlations were found between the methylation levels of these genes and clinical indicators. This suggests that methylation of negative JAK/STAT regulators plays a minor role in the course of ET [117].

Zhang M.Y. et al. have demonstrated that methylation of the *SOCS1*, *SOCS2*, *SOCS3*, and *CISH* genes is not common in Ph(-) myeloproliferative neoplasms. They also determined the methylation level of the *SHP1* gene, which varied in both the studied cell lines and the examined bone marrow samples from patients diagnosed with MPN; as such, its role in the pathogenesis of the disease requires further investigation [118].

Dursun Torlak E. et al. attempted to demonstrate differences in the methylation pattern in patients with PMF depending on the type of driver mutation; however, their results indicated that the mutation profile itself did not significantly affect methylation. Instead, the methylation profile varied depending on the cellular composition, and was found to be suitable for differentiating between different myeloid diseases [119].

None of the articles covered in this review identified a significant role of DNA methylation in the development of ET, and the only abnormalities with a proven effect on the risk of disease progression were SNP and hypermethylation of the *PTP1B* gene promoters [115]. Due to the limited number of studies on methylation profiles conducted in groups of ET patients to date, the precise role of methylation in the pathogenesis of the disease remains unclear.

## 9. Histone Post-Translational Modifications

The primary function of histone proteins is chromatin packaging. Through numerous post-translational modifications—such as phosphorylation, acetylation and deacetylation, methylation and demethylation, ADP-ribosylation, and ubiquitination—histones interact with individual transcription factors, thus regulating gene expression; modifying chromatin condensation; and influencing cellular processes such as replication, apoptosis, and DNA repair [120].

Histone modifications are one of the epigenetic processes seen in ET. Mutant JAK2, in addition to its role in cytokine signaling, exhibits epigenetic activity in the nucleus, where it phosphorylates histone H3 at Y41, consequently blocking heterochromatin protein 1α binding and deregulating the transcription of genes involved in proliferation, differentiation, and DNA repair. JAK2 also phosphorylates the arginine methyltransferase PRMT5, disrupting its interaction with MEP50 and reducing methylation of histones H2A and H4, thereby promoting gene derepression. Furthermore, the *N*-terminal tails of histones—particularly H3 and H4—are subject to acetylation, methylation, and phosphorylation, which regulate chromatin structure and transcriptional activity. The imbalance between histone acetyltransferases and deacetylases (HDACs) observed in ET leads to sustained activation of proliferative genes, highlighting the importance of epigenetics in the pathogenesis of the disease [114].

Wang J.C. et al. assessed histone deacetylase (HDAC) activity in patients with MPN compared to healthy controls, and found that this activity was significantly higher in samples from patients with myelofibrosis compared to those from patients with other MPNs and healthy controls. This suggests that HDACs may play a role in the progression of ET and PV to MF, and suggests the potential of HDAC inhibitors in the treatment of patients with myelofibrosis [121].

Dunphy K. et al. summarized the current knowledge (as of 2021) regarding the potential use of post-translational modification analyses for diagnosis, prognosis, and therapy in patients with MPN. It was emphasized that class IV histone deacetylase inhibitors—namely, HDAC11 and, to a lesser extent, HDAC6—stimulate proliferation and abnormal hematopoiesis in JAK2-positive MPN and, thus, may represent a promising therapeutic target in the future [122].

In this paper, we previously described the most common gene mutations affecting the epigenetic profiles of patients with essential thrombocythemia. Among them, a mutation in the *ASXL1* gene was described, which—along with mutations in another gene, *EZH2*—affects histone modification. The latter mutation leads to synthesis of the catalytic repressive complex Polycomb 2, which is responsible for activation of histone methyltransferase H3K27me3 that, in turn, leads to gene silencing. When *EZH2* mutations occur in patients with ET, they are associated with higher mortality and myofibrotic and blastic transformation [123].

## 10. Conclusions

Previous research has indicated that patients with ET constitute a highly heterogeneous group, with the course and prognosis of their disease largely depending on the presence of specific mutation profiles. Therefore, it is necessary to accurately categorize these patients and tailor personalized diagnostic and therapeutic approaches.

Significant progress in genetic diagnostics has been made in recent years, and sensitive methods for the qualitative and quantitative detection of driver mutations, advanced NGS techniques, and approaches to identify specific epigenetic abnormalities are now available. The roles of these abnormalities and their impacts on the course of the disease are not yet fully understood, and further research involving diverse patient groups is required. It is also crucial to select the best material for studying genetic and epigenetic abnormalities in ET. To date, relevant studies have used genetic material obtained from various sources, including blood serum cells, bone marrow, or directly from platelets, making it difficult to compare the conclusions from individual studies. In the studies conducted so far, noticeable differences in the expression of selected miRNAs in peripheral blood from patients with myeloproliferative neoplasms compared to healthy individuals have been reported. Similar differences have also been observed when examining miRNAs in platelets from patients with ET compared to controls. Notably, individual miRNAs have been shown to play roles in the maturation and functioning of platelets, which may be crucial in the pathogenesis of ET.

Methylation changes in genes regulating the JAK/STAT pathway—including *PTP1B*, *SOCS1*, *SOCS3*, *PTPN6*, and *SHP1*—observed in MPN patients do not clearly correlate with the clinical profile, nor the type of driver mutation. Therefore, data regarding their exact biological significance remain equivocal and further research is needed to clarify their roles. An increase in differentially methylated regions has been observed in patients who transformed to AML compared to those in the chronic phase, potentially suggesting that altered DNA methylation is involved in disease progression. Another interesting observation is that HDAC activity was significantly higher in individuals with bone marrow fibrosis compared to those with other MPNs. This suggests the participation of HDACs in the progression of ET and PV to MF, informing a potential target in future therapies.

Identifying and describing specific relationships among individual miRNAs, driver mutations, and disease phenotypes may enable more accurate diagnoses and more precise predictions regarding the course and prognosis of ET. Previous progress in genetics and epigenetics research has helped us to better understand the pathogenesis of ET, develop new diagnostic methods, assess risk, and create new therapeutic options. Historically, treatment for essential thrombocythemia was limited to thromboembolism prophylaxis, followed by addition of the cytoreductive drug hydroxycarbamide. When genetic changes influencing disease progression were discovered, the goal shifted to reducing the expression of the mutation, leading to the introduction of drugs that reduce the VAF—initially interferon, and later drugs that inhibit JAK/STAT activity, including ruxolitinib. A future therapeutic goal may be influencing the epigenetic changes involved in disease progression. However, interfering with the JAK/STAT pathway affects not only cancer cells but also healthy cells without the driver mutation, leading to numerous side effects. This makes the development of more targeted therapies for patients with ET an urgent task. In the future, we may be able to develop targeted treatments that are tailored specifically to the molecular model of a given type of MPN.

The research conducted to date on the influence of miRNAs in ET suggests that these molecules play significant roles in the pathogenesis of the disease; however, many aspects of these potential roles remain unclear. Further research is needed to understand the impacts of miRNAs on the expression of driver mutations, differences in hematopoiesis between various MPNs, and the risk of disease progression. Understanding these processes could enable the precise identification of specific disease subtypes and the development of new therapies aimed at changing the course of—or even completely curing—the disease.

In summary, patients with essential thrombocythemia constitute an extremely heterogeneous group. With the advances in genetics and epigenetics made in recent years, our understanding of the pathogenesis and factors influencing this population has evolved considerably. In the future, researchers should focus on obtaining a more detailed understanding of the impacts of specific genetic and epigenetic changes occurring in ET, which may allow for the development of targeted, individualized therapies that can be expected to significantly improve patients’ quality of life, extend progression-free survival, and perhaps even completely and permanently halt the progression of the disease.

## Figures and Tables

**Table 1:** Driver mutations in ET: frequency, mechanism of action, influence on the disease phenotype.

Driver Mutation	Frequency	Mechanism	Phenotype
*JAK2*	(50–60%)	Mutation located in JH2 inhibits the pseudokinase’s inhibitory function on JH1. Continuous JAK2 activation. Dysregulation of the JAK/STAT, PI3K/AKT and MAPK pathways. Constant activation of the MPL (TOPR) receptor	Higher median age at diagnosis More frequently found in women Higher hemoglobin and leukocyte levels The most prone to thromboembolic complications Higher rate of serious bleeding events
*CALR*	(20–25%)	Mutated calreticulin is secreted from the ER, binds to the MPL or TOPR receptor and activates it by dimerization resulting in continuously activated *JAK2* independent of thrombopoietin	Lower median age at diagnosis Higher platelet count Lower risk of thromboembolism Increased risk of progression to MF
*MPL*	(3–4%)	Point mutation in exon 10 resulting in disrupting the receptor’s transmembrane region. Constitutive activation of TOPR and, consequently, the JAK-STAT pathway, promoting TPO-independent platelet proliferation	Higher platelet count Increased risk of progression to MF Higher rate of serious bleeding events

Abbreviations: MF—myelofibrosis, JH1—pseudokinase domain, JH2—C-terminal kinase domain, ER—endoplasmic reticulum.

**Table 2:** The influence of additional mutations on the pathogenesis, phenotype, and prognosis of ET.

Significance	Mutations
Regulating epigenome changes	*ASXL1*, *TET2*, *EZH2*, *IDH1*, *IDH2*, *DNMT3A* [5]
RNA splicing	*SRSF2*, *U2AF1*, *ZRSF2*, *SF3B1 SRSF2*, *U2AF1*, *ZRSF2*, *SF3B1* [5]
Transcription	*TP53*, *IKZF1*, *NF-E2*, *CUX1* [5]
Increased platelet count	*SF3B1* [60]
Enlarged spleen	*ASXL1* [60], *CBL* [38]
Increased the risk of thrombosis	*TET2* [60]
Increased risk of bleeding	*ASXL1* [71,72]
Increased cardiovascular risk	*DNMT3A*, *TET2*, *ASXL1* [61]
Negative impacts on OS	*IDH2*, *EZH2*, *SH2B3* [63] *SF3B1*, *SRSF2*, *EZH1*, *U2AF1* [67]
Negative impact on LFS	*SRSF2*, *EZH2*, *TP53*, *U2AF1*, *RUNX1* [67] *IDH1/2* [77]
Negative impact on MFFS	*SF3B1*, *U2AF1*, *SF3B1*, *SRSF2* [67]

Abbreviations: OS—overall survival, LFS—leukemia-free survival, MFFS—myelofibrosis-free survival.

**Table 3:** Key exosomal microRNAs in ET: expression, targets, clinical relevance and the material on which the study was conducted.

Key Exosomal microRNAs	Expression in ET	Key Targets/Pathways	Effect	Tested Material
miR-490	Lower	*DAAM1*	Greater thrombotic risk	Platelets [99]
miR-28	Higher	*MPL* *E2F6* *ERK2*	Constitutive JAK2/STAT activation Stimulation of megakaryopoiesis	Platelets [101]
miR-155-5p	Higher	*JARID2*	Stimulation of megakaryopoiesis	CD34+ cells [86]
miR-143	Higher	unknown	*JAK2* VAF growth Stimulation of megakaryopoiesis	Granulocytes [103]
miR-146a	Lower	NF-κB	Risk factor for progression to MF (independent of *JAK2* mutation)	Granulocytes Peripheral whole blood [104]
miR-203 miR-221	Higher	*SOCS3*	Constitutive JAK2/STAT activation	Platelets [106]
miR-375	Lower	*JAK2*	Constitutive JAK2/STAT activation Can be modulated by HDAC inhibitors—a therapeutic target	Bone marrow [107]
miR-101	Higher	*SOCS2*	Constitutive JAK2/STAT activation	Granulocytes Mononuclear cells [110]

Abbreviations: ET—Essential Thrombocythemia, HDAC—histone deacetylase.

## Data Availability

No new data were created or analyzed in this study. Data sharing is not applicable to this article.
